# 2-Oxo-2*H*-chromen-4-yl 3,3-di­methyl­butano­ate

**DOI:** 10.1107/S2414314624004942

**Published:** 2024-05-31

**Authors:** Valentin Bationo, Eric Ziki, Charles Bavouma Sombié, Rasmané Semdé, Abdoulaye Djandé

**Affiliations:** a Laboratory of Molecular Chemistry and Materials, Research Team: Organic Chemistry and Phytochemistry, University Joseph KI-ZERBO, 03 BP 7021 Ouagadougou 03, Burkina Faso; b Laboratory of Material, Sciences, Environnement and Solar Energy, Research Team: Crystallography and Molecular Physics, University Félix Houphouët-Boigny, 08 BP 582 Abidjan, Ivory Coast; c Laboratory of Drug Development, Center of Training Reasearch and Expertise in Pharmaceutical Sciences (CFOREM), University Joseph KI-ZERBO, 03 BP 7021 Ouagadougou 30, Burkina Faso; University of Aberdeen, United Kingdom

**Keywords:** crystal structure, coumarin, Hirshfeld surface

## Abstract

In the crystal of the title compound, C_15_H_16_O_4_, the mol­ecules are connected through C—H⋯O hydrogen bonds, generating [100] chains, which are crosslinked by weak π–π stacking inter­actions.

## Abstract 2

In the crystal of the title compound, the mol­ecules are connected through C—H⋯O hydrogen bonds, generating [100] chains, which are crosslinked by weak π–π stacking inter­actions.

## Graphical abstract

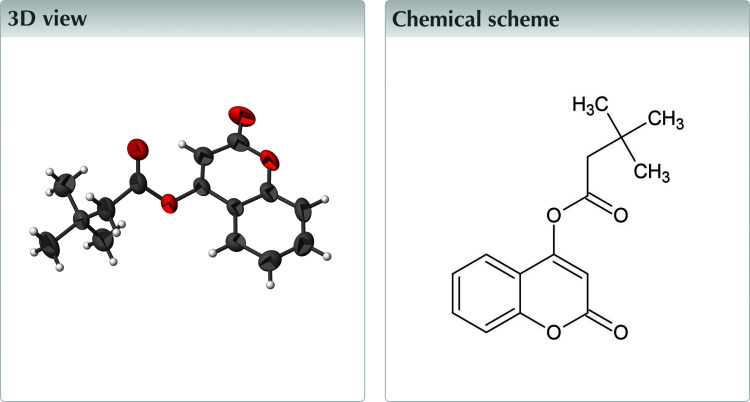

## Structure description

Coumarin derivatives show various biological activities such as anti­cancer (Lacy & O’Kennedy, 2004 ▸; Kostova, 2005 ▸), anti-inflammatory (Todeschini *et al.*, 1998 ▸) and anti­viral (Borges *et al.*, 2005 ▸) properties. As part of our ongoing studies in this area (Ziki *et al.*, 2017 ▸), we now describe the synthesis and structure of the title compound, C_15_H_16_O_4_.

**Figure 1:**
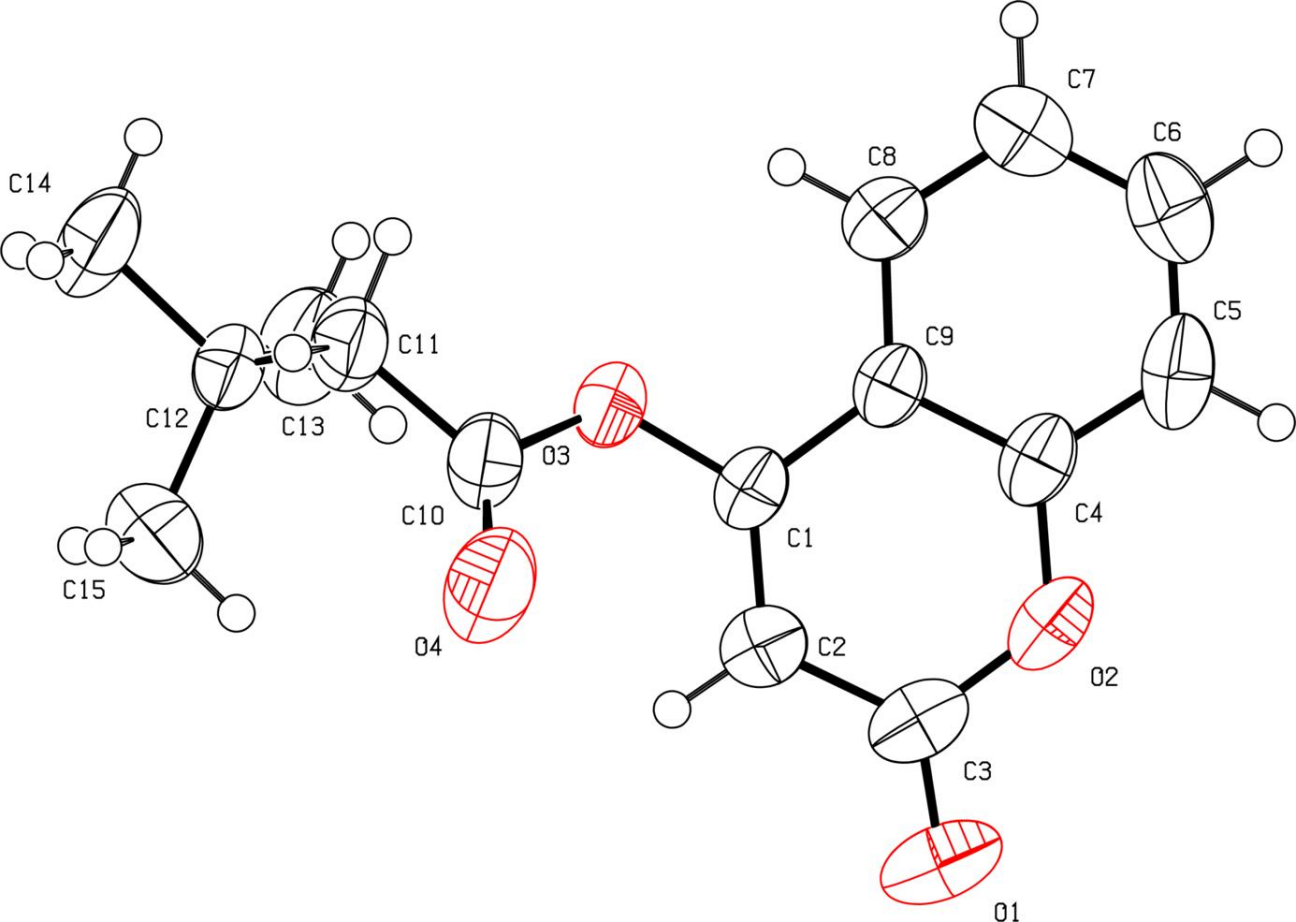
The mol­ecular structure of the title compound with displacement ellipsoids drawn at the 50% probability level.

**Figure 2:**
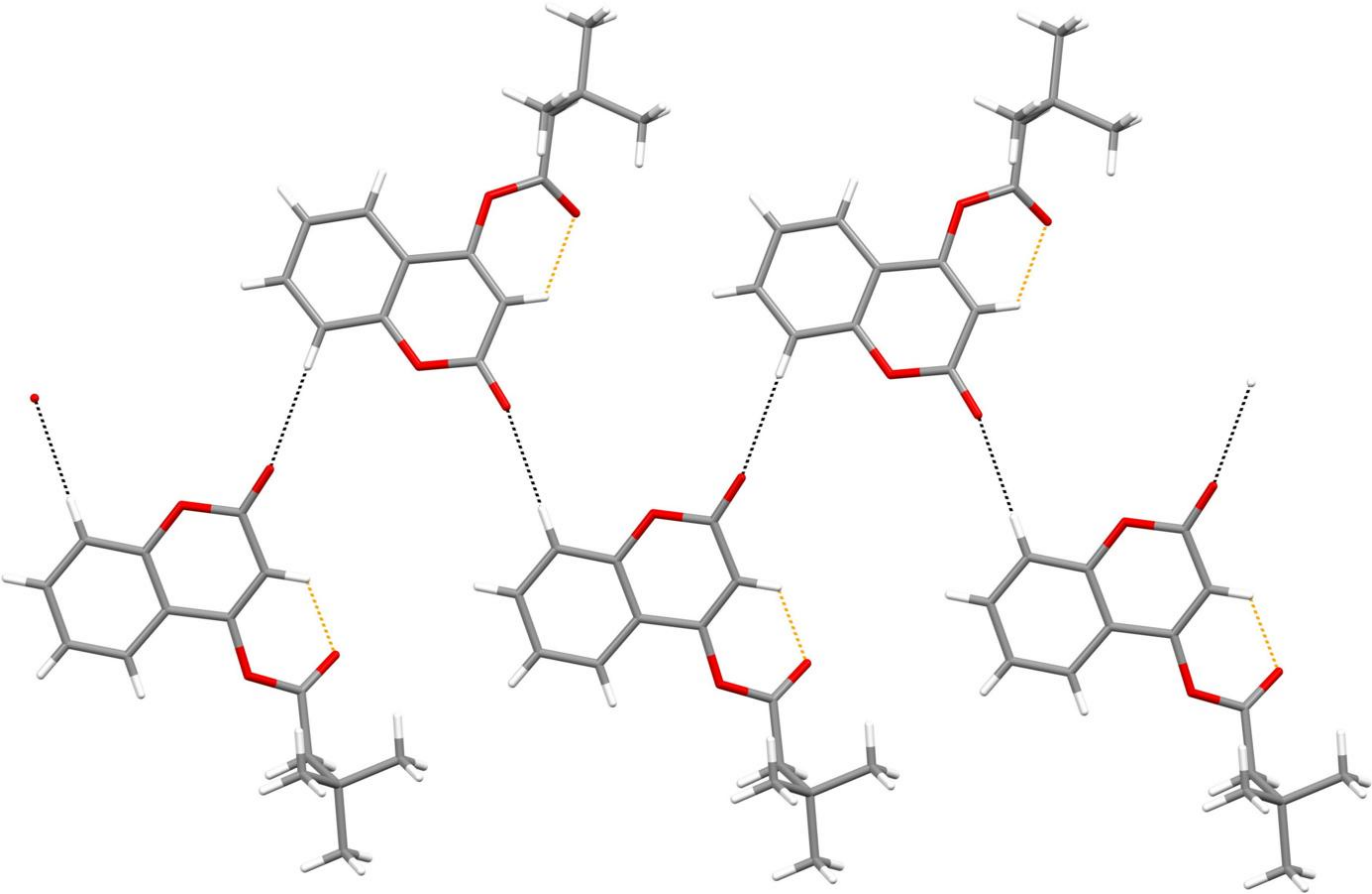
Part of a [100] hydrogen-bonded chain in the extended structure of the title compound. The inter­molecular hydrogen bonds are shown as black dashed lines and the short intra­molecular contacts as orange dashed lines.

As expected, the coumarin ring system is almost planar (r.m.s deviation = 0.025 Å) and oriented at an angle of 56.24 (18)° with the C10/C11/O3/O4 butano­ate moiety (Fig. 1 ▸). The C1—C2 [1.332 (2) Å] and C2—C3 [1.446 (3) Å] bond lengths are shorter and longer, respectively, than those excepted for an aromatic C—C bond (1.38 Å). This suggests that the C1—C2 bond has significant double-bond character, as seen in other coumarin derivatives (*e.g.*, Gomes *et al.*, 2016 ▸). A short intra­molecular C2—H2⋯O4 contact occurs (Table 1 ▸). If this is regarded as a directional bond, an *S*(6) ring is generated. In the extended structure, the mol­ecules are linked by weak C5—H1⋯O1 hydrogen bonds, generating [100] *C*(6) chains (Fig. 2 ▸). Weak aromatic π–π stacking between the C4–C9 rings [centroid–centroid separation = 3.8987 (12) Å, tilt angle = 10.08 (10)°] crosslink the chains in the [001] direction.

**Figure 3:**
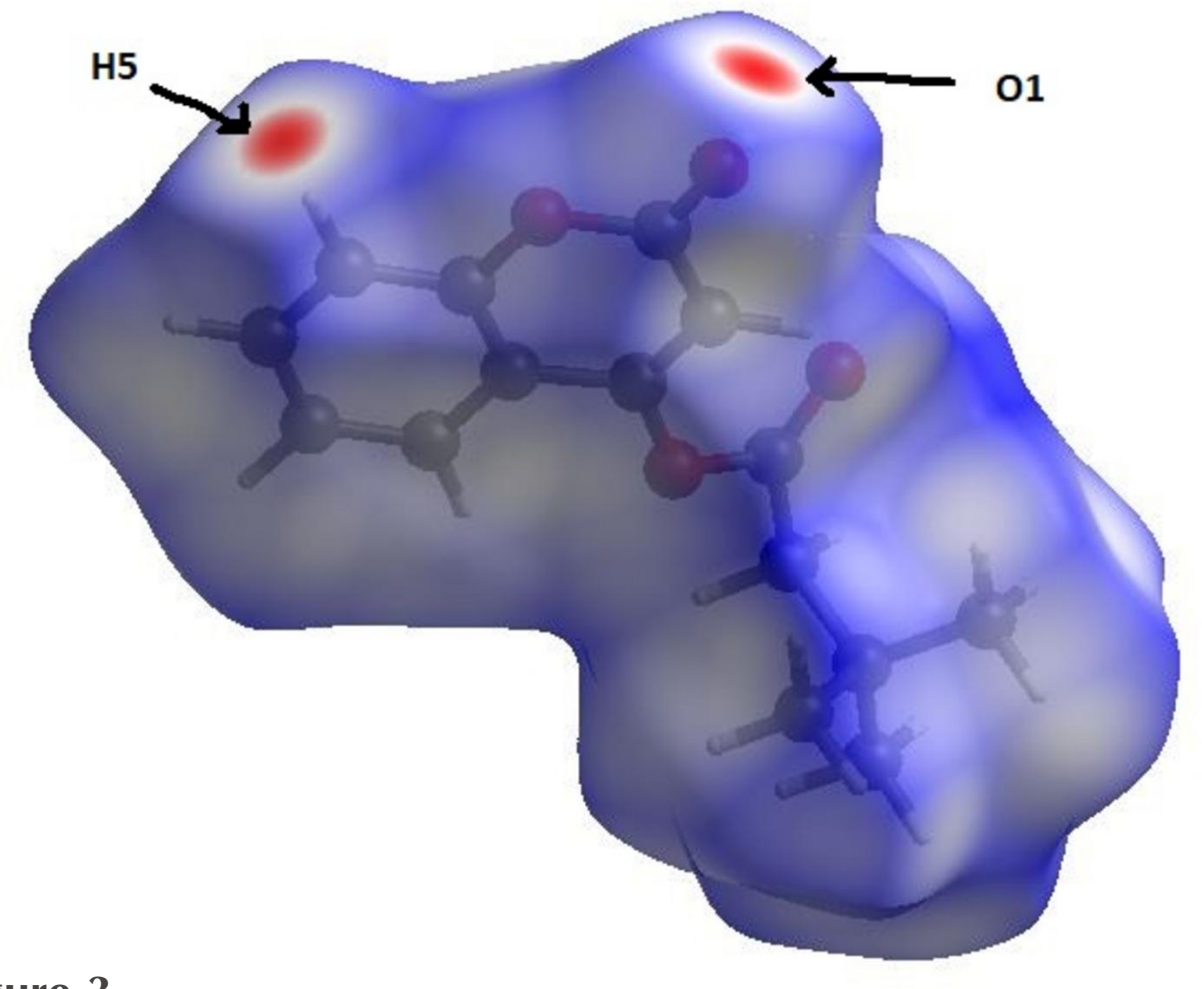
A view of the Hirshfeld surface mapped over *d*_norm_. The short contact points (red) are labelled to indicate the atoms participating in the inter­molecular inter­actions.

**Figure 4:**
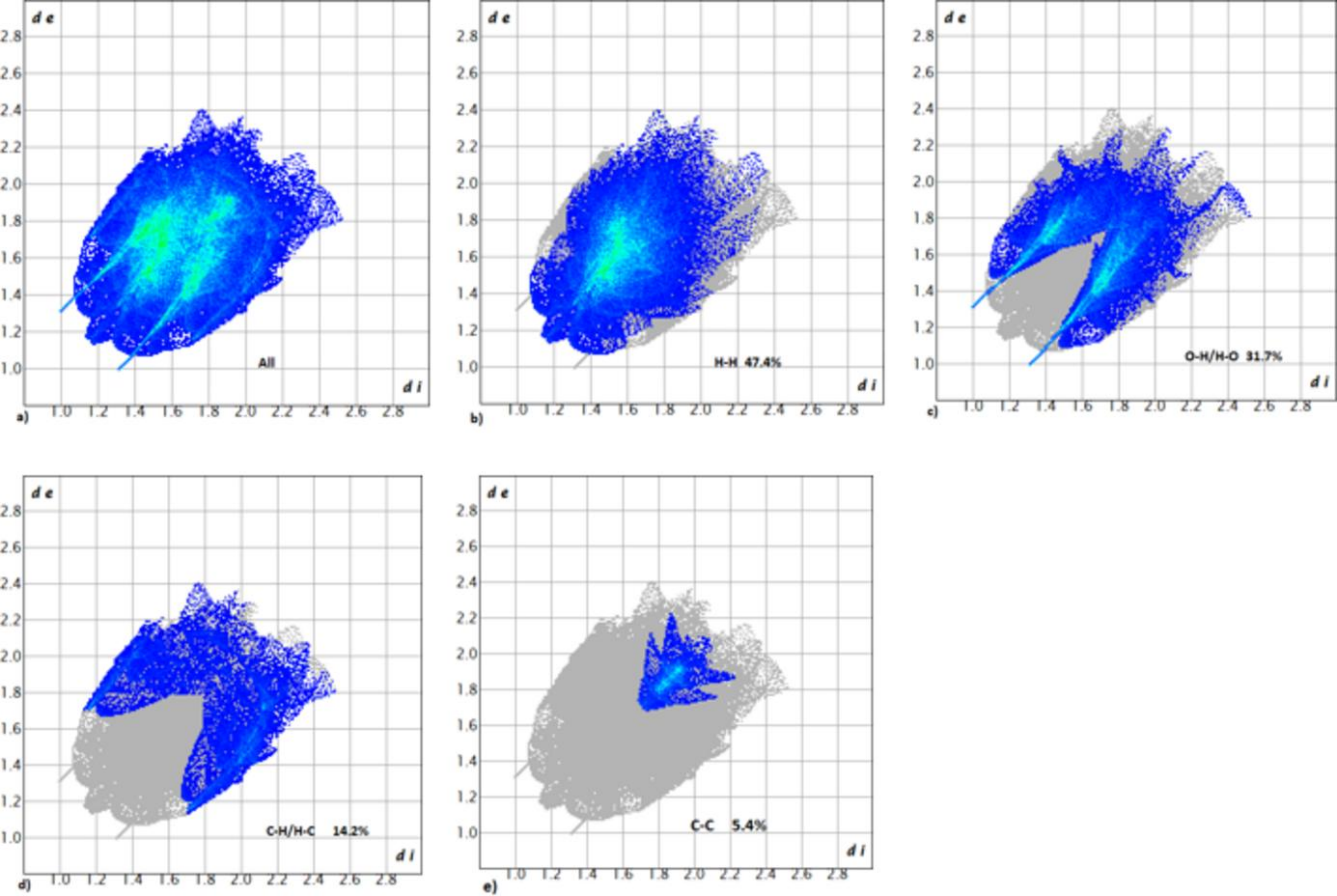
Two-dimensional fingerprint plots: (*a*) overall, and delineated into contributions from different contacts: (*b*) H⋯H, (*c*) H⋯O/O⋯H, (*d*) H⋯C/C⋯H and (*e*) C⋯C.

The only red spots (close contacts) on the Hirshfeld surface of the title compound generated by *CrystalExplorer17* (Spackman *et al.*, 2021 ▸) are associated with the hydrogen-bond donor H5 and acceptor O1 atoms noted above (Fig. 3 ▸). The two-dimensional fingerprint plots (Fig. 4 ▸ *a*–*e*) show that the main contributions to the Hirshfeld surface are H⋯H, H⋯O/O⋯H, H⋯C/C⋯H and C⋯C contacts, which contribute 47.4, 31.7, 14.2 and 5.4%, respectively.

## Synthesis and crystallization

In a 100 ml round-necked flask topped with a water condenser were introduced successively: dried diethyl ether (16 ml), *tert*-butyl­acetyl chloride (0.90 ml, 6.2 mmol) and dried pyridine (2.31 ml, 4.7 molar equivalents). With vigorous stirring, 4-hy­droxy­coumarin (1.00 g; 6.17 mmol) was added in small portions over 30 min. The reaction mixture was left stirring at room temperature for 3 h. The mixture was then poured in a separating funnel containing 40 ml of chloro­form and washed with diluted hydro­chloric acid solution until the pH was 2–3. The organic phase was extracted, washed with water to neutrality, dried over MgSO_4_ and the solvent removed. The resulting precipitate was filtered off with suction, washed with *n*-pentane and recrystallized from acetone solution to obtain colourless prisms of the title compound: yield 63%; m.p. 430–431 K

## Refinement

Crystal data, data collection and structure refinement details are summarized in Table 2 ▸.

## Supplementary Material

Crystal structure: contains datablock(s) I. DOI: 10.1107/S2414314624004942/hb4474sup1.cif

Structure factors: contains datablock(s) I. DOI: 10.1107/S2414314624004942/hb4474Isup2.hkl

Supporting information file. DOI: 10.1107/S2414314624004942/hb4474Isup3.cml

CCDC reference: 2336029

Additional supporting information:  crystallographic information; 3D view; checkCIF report

## Figures and Tables

**Table 1:** Hydrogen-bond geometry (Å, °)

*D*—H⋯*A*	*D*—H	H⋯*A*	*D*⋯*A*	*D*—H⋯*A*
C2—H2⋯O4	0.93	2.44	2.847 (3)	107
C5—H5⋯O1^i^	0.93	2.48	3.405 (2)	176

Symmetry code: (i) 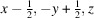 .

**Table 2:** Experimental details

Crystal data	
Chemical formula	C_15_H_16_O_4_
*M*_r_	260.28
Crystal system, space group	Orthorhombic, *P* *n* *a*2_1_
Temperature (K)	295
*a*, *b*, *c* (Å)	10.6769 (3), 17.9611 (5), 7.0266 (2)
*V* (Å^3^)	1347.48 (7)
*Z*	4
Radiation type	Cu *K*α
μ (mm^−1^)	0.76
Crystal size (mm)	0.32 × 0.18 × 0.16

Data collection	
Diffractometer	SuperNova, Dual, Cu at home/near, AtlasS2
Absorption correction	Multi-scan (*CrysAlis PRO*; Rigaku OD, 2023 ▸)
*T*_min_, *T*_max_	0.829, 1.000
No. of measured, independent and observed [*I* > 2σ(*I*)] reflections	9694, 2249, 2133
*R*_int_	0.020
(sin θ/λ)_max_ (Å^−1^)	0.618

Refinement	
*R*[*F* ^2^ > 2σ(*F* ^2^)], *wR*(*F* ^2^), *S*	0.031, 0.084, 1.05
No. of reflections	2249
No. of parameters	176
No. of restraints	1
H-atom treatment	H-atom parameters constrained
Δρ_max_, Δρ_min_ (e Å^−3^)	0.11, −0.13
Absolute structure	Refined as an inversion twin.
Absolute structure parameter	0.5 (3)

Computer programs: *CrysAlis PRO* (Rigaku OD, 2023 ▸), *SHELXT2015* (Sheldrick, 2015*a* ▸), *SHELXL2013* (Sheldrick, 2015*b* ▸), *ORTEP-3* fro Windows (Farrugia, 2012 ▸) and *publCIF* (Westrip, 2010 ▸).

## full crystallographic data

### Crystal data

**Table:**

C_15_H_16_O_4_	*D*_x_ = 1.283 Mg m^−^^3^
*M**_r_* = 260.28	Melting point: 430 K
Orthorhombic, *P**n**a*2_1_	Cu *K*α radiation, λ = 1.54184 Å
*a* = 10.6769 (3) Å	Cell parameters from 6044 reflections
*b* = 17.9611 (5) Å	θ = 4.8–72.4°
*c* = 7.0266 (2) Å	µ = 0.76 mm^−^^1^
*V* = 1347.48 (7) Å^3^	*T* = 295 K
*Z* = 4	Prism, colourless
*F*(000) = 552	0.32 × 0.18 × 0.16 mm

### Data collection

**Table:**

SuperNova, Dual, Cu at home/near, AtlasS2 diffractometer	2133 reflections with *I* > 2σ(*I*)
Radiation source: micro-focus sealed X-ray tube; SuperNova (Cu) X-ray Source	*R*_int_ = 0.020
Mirror monochromator	θ_max_ = 72.4°, θ_min_ = 4.8°
ω scan	*h* = −12→13
Absorption correction: multi-scan (CrysAlis PRO; Rigaku OD, 2023)	*k* = −22→21
*T*_min_ = 0.829, *T*_max_ = 1.000	*l* = −8→7
9694 measured reflections	2249 standard reflections every 25 min
2249 independent reflections	

### Refinement

**Table:**

Refinement on *F*^2^	Secondary atom site location: difference Fourier map
Least-squares matrix: full	Hydrogen site location: inferred from neighbouring sites
*R*[*F*^2^ > 2σ(*F*^2^)] = 0.031	H-atom parameters constrained
*wR*(*F*^2^) = 0.084	*w* = 1/[σ^2^(*F*_o_^2^) + (0.049*P*)^2^ + 0.0768*P*] where *P* = (*F*_o_^2^ + 2*F*_c_^2^)/3
*S* = 1.05	(Δ/σ)_max_ < 0.001
2249 reflections	Δρ_max_ = 0.11 e Å^−^^3^
176 parameters	Δρ_min_ = −0.13 e Å^−^^3^
1 restraint	Absolute structure: Refined as an inversion twin.
Primary atom site location: structure-invariant direct methods	Absolute structure parameter: 0.5 (3)

### Special details

**Table:**

Geometry. All esds (except the esd in the dihedral angle between two l.s. planes) are estimated using the full covariance matrix. The cell esds are taken into account individually in the estimation of esds in distances, angles and torsion angles; correlations between esds in cell parameters are only used when they are defined by crystal symmetry. An approximate (isotropic) treatment of cell esds is used for estimating esds involving l.s. planes.
Refinement. Refined as a 2-component inversion twin.

### Fractional atomic coordinates and isotropic or equivalent isotropic displacement parameters (Å^2^)

**Table:**

	*x*	*y*	*z*	*U*_iso_*/*U*_eq_	
O1	0.81314 (18)	0.29193 (8)	0.4568 (3)	0.0877 (6)	
O2	0.62460 (14)	0.33944 (6)	0.4897 (2)	0.0631 (4)	
O3	0.76506 (11)	0.55295 (6)	0.5416 (2)	0.0578 (4)	
O4	0.93542 (18)	0.52915 (9)	0.7209 (4)	0.0924 (7)	
C1	0.72707 (16)	0.47995 (8)	0.5255 (3)	0.0468 (4)	
C2	0.80284 (18)	0.42179 (10)	0.5013 (4)	0.0569 (4)	
H2	0.8891	0.4290	0.4974	0.068*	
C3	0.7522 (2)	0.34755 (10)	0.4809 (3)	0.0615 (5)	
C4	0.54603 (17)	0.39958 (9)	0.5092 (3)	0.0504 (4)	
C5	0.4183 (2)	0.38581 (12)	0.5105 (3)	0.0650 (5)	
H5	0.3882	0.3373	0.5014	0.078*	
C6	0.33703 (19)	0.44444 (14)	0.5253 (3)	0.0673 (6)	
H6	0.2512	0.4356	0.5262	0.081*	
C7	0.38128 (18)	0.51700 (12)	0.5390 (3)	0.0623 (5)	
H7	0.3252	0.5565	0.5470	0.075*	
C8	0.50808 (17)	0.53037 (10)	0.5406 (3)	0.0515 (4)	
H8	0.5375	0.5789	0.5509	0.062*	
C9	0.59267 (16)	0.47158 (8)	0.5268 (3)	0.0435 (4)	
C10	0.87001 (19)	0.57316 (12)	0.6437 (4)	0.0583 (5)	
C11	0.8867 (2)	0.65593 (11)	0.6425 (4)	0.0604 (5)	
H11A	0.9275	0.6707	0.7599	0.072*	
H11B	0.8046	0.6792	0.6401	0.072*	
C12	0.96367 (16)	0.68573 (9)	0.4739 (3)	0.0516 (4)	
C13	0.9024 (3)	0.66854 (15)	0.2840 (4)	0.0798 (7)	
H13A	0.8958	0.6156	0.2684	0.120*	
H13B	0.9523	0.6889	0.1830	0.120*	
H13C	0.8203	0.6903	0.2806	0.120*	
C14	0.9745 (3)	0.77005 (12)	0.5023 (6)	0.0868 (8)	
H14A	0.8922	0.7915	0.5070	0.130*	
H14B	1.0204	0.7914	0.3983	0.130*	
H14C	1.0175	0.7801	0.6195	0.130*	
C15	1.0947 (2)	0.65202 (14)	0.4765 (5)	0.0739 (6)	
H15A	1.1348	0.6636	0.5952	0.111*	
H15B	1.1430	0.6722	0.3735	0.111*	
H15C	1.0889	0.5990	0.4626	0.111*	

### Atomic displacement parameters (Å^2^)

**Table:**

	*U*^11^	*U*^22^	*U*^33^	*U*^12^	*U*^13^	*U*^23^
O1	0.1116 (13)	0.0485 (8)	0.1030 (14)	0.0254 (8)	−0.0004 (12)	−0.0058 (9)
O2	0.0819 (9)	0.0344 (6)	0.0732 (10)	−0.0071 (5)	−0.0018 (9)	0.0023 (7)
O3	0.0523 (6)	0.0387 (6)	0.0824 (10)	−0.0079 (5)	−0.0064 (7)	0.0043 (6)
O4	0.0924 (12)	0.0634 (9)	0.1214 (18)	−0.0185 (9)	−0.0473 (12)	0.0232 (10)
C1	0.0538 (9)	0.0364 (8)	0.0503 (10)	−0.0053 (6)	0.0001 (9)	0.0039 (8)
C2	0.0570 (9)	0.0482 (9)	0.0654 (12)	0.0032 (7)	0.0014 (10)	0.0019 (10)
C3	0.0812 (13)	0.0434 (9)	0.0598 (13)	0.0092 (9)	−0.0012 (12)	0.0017 (9)
C4	0.0656 (10)	0.0421 (8)	0.0436 (9)	−0.0100 (7)	−0.0008 (9)	0.0045 (8)
C5	0.0738 (12)	0.0637 (11)	0.0574 (12)	−0.0302 (10)	−0.0038 (11)	0.0092 (11)
C6	0.0533 (10)	0.0923 (15)	0.0563 (12)	−0.0153 (10)	0.0005 (11)	0.0094 (13)
C7	0.0545 (10)	0.0762 (13)	0.0562 (11)	0.0065 (9)	0.0010 (10)	0.0033 (11)
C8	0.0579 (9)	0.0468 (9)	0.0498 (10)	0.0003 (7)	−0.0009 (9)	0.0014 (8)
C9	0.0527 (8)	0.0381 (7)	0.0397 (8)	−0.0065 (6)	0.0001 (8)	0.0039 (7)
C10	0.0575 (10)	0.0514 (10)	0.0660 (12)	−0.0137 (9)	−0.0017 (10)	0.0029 (10)
C11	0.0597 (11)	0.0497 (10)	0.0716 (13)	−0.0117 (8)	0.0073 (10)	−0.0099 (10)
C12	0.0525 (9)	0.0398 (8)	0.0626 (12)	−0.0086 (7)	−0.0034 (9)	−0.0019 (8)
C13	0.0913 (17)	0.0747 (16)	0.0733 (16)	−0.0182 (13)	−0.0236 (13)	0.0109 (13)
C14	0.1012 (16)	0.0441 (10)	0.115 (2)	−0.0192 (10)	0.0060 (19)	−0.0045 (14)
C15	0.0576 (11)	0.0847 (14)	0.0793 (16)	−0.0015 (10)	0.0018 (12)	−0.0032 (13)

### Geometric parameters (Å, º)

**Table:**

O1—C3	1.204 (2)	C8—C9	1.393 (2)
O2—C3	1.371 (3)	C8—H8	0.9300
O2—C4	1.374 (2)	C10—C11	1.497 (3)
O3—C1	1.3771 (19)	C11—C12	1.538 (3)
O3—C10	1.379 (2)	C11—H11A	0.9700
O4—C10	1.186 (3)	C11—H11B	0.9700
C1—C2	1.332 (2)	C12—C13	1.518 (3)
C1—C9	1.443 (2)	C12—C15	1.525 (3)
C2—C3	1.446 (3)	C12—C14	1.532 (3)
C2—H2	0.9300	C13—H13A	0.9600
C4—C5	1.386 (3)	C13—H13B	0.9600
C4—C9	1.391 (2)	C13—H13C	0.9600
C5—C6	1.368 (3)	C14—H14A	0.9600
C5—H5	0.9300	C14—H14B	0.9600
C6—C7	1.390 (3)	C14—H14C	0.9600
C6—H6	0.9300	C15—H15A	0.9600
C7—C8	1.375 (3)	C15—H15B	0.9600
C7—H7	0.9300	C15—H15C	0.9600
			
C3—O2—C4	121.82 (13)	O3—C10—C11	110.79 (18)
C1—O3—C10	122.19 (15)	C10—C11—C12	114.42 (18)
C2—C1—O3	125.33 (16)	C10—C11—H11A	108.7
C2—C1—C9	121.53 (15)	C12—C11—H11A	108.7
O3—C1—C9	113.06 (14)	C10—C11—H11B	108.7
C1—C2—C3	120.57 (18)	C12—C11—H11B	108.7
C1—C2—H2	119.7	H11A—C11—H11B	107.6
C3—C2—H2	119.7	C13—C12—C15	109.0 (2)
O1—C3—O2	117.08 (19)	C13—C12—C14	110.4 (2)
O1—C3—C2	125.2 (2)	C15—C12—C14	108.79 (17)
O2—C3—C2	117.71 (16)	C13—C12—C11	112.06 (17)
O2—C4—C5	117.45 (16)	C15—C12—C11	110.08 (19)
O2—C4—C9	121.39 (16)	C14—C12—C11	106.50 (19)
C5—C4—C9	121.16 (18)	C12—C13—H13A	109.5
C6—C5—C4	119.15 (18)	C12—C13—H13B	109.5
C6—C5—H5	120.4	H13A—C13—H13B	109.5
C4—C5—H5	120.4	C12—C13—H13C	109.5
C5—C6—C7	120.76 (18)	H13A—C13—H13C	109.5
C5—C6—H6	119.6	H13B—C13—H13C	109.5
C7—C6—H6	119.6	C12—C14—H14A	109.5
C8—C7—C6	119.95 (19)	C12—C14—H14B	109.5
C8—C7—H7	120.0	H14A—C14—H14B	109.5
C6—C7—H7	120.0	C12—C14—H14C	109.5
C7—C8—C9	120.36 (17)	H14A—C14—H14C	109.5
C7—C8—H8	119.8	H14B—C14—H14C	109.5
C9—C8—H8	119.8	C12—C15—H15A	109.5
C4—C9—C8	118.61 (16)	C12—C15—H15B	109.5
C4—C9—C1	116.89 (15)	H15A—C15—H15B	109.5
C8—C9—C1	124.49 (15)	C12—C15—H15C	109.5
O4—C10—O3	122.75 (19)	H15A—C15—H15C	109.5
O4—C10—C11	126.5 (2)	H15B—C15—H15C	109.5
			
C10—O3—C1—C2	−40.0 (3)	C5—C4—C9—C8	−1.7 (3)
C10—O3—C1—C9	143.23 (18)	O2—C4—C9—C1	−0.6 (3)
O3—C1—C2—C3	−178.33 (19)	C5—C4—C9—C1	179.5 (2)
C9—C1—C2—C3	−1.8 (3)	C7—C8—C9—C4	0.7 (3)
C4—O2—C3—O1	−177.4 (2)	C7—C8—C9—C1	179.4 (2)
C4—O2—C3—C2	2.6 (3)	C2—C1—C9—C4	2.5 (3)
C1—C2—C3—O1	179.2 (2)	O3—C1—C9—C4	179.41 (17)
C1—C2—C3—O2	−0.7 (3)	C2—C1—C9—C8	−176.2 (2)
C3—O2—C4—C5	178.0 (2)	O3—C1—C9—C8	0.7 (3)
C3—O2—C4—C9	−1.9 (3)	C1—O3—C10—O4	1.4 (4)
O2—C4—C5—C6	−178.5 (2)	C1—O3—C10—C11	−178.15 (18)
C9—C4—C5—C6	1.4 (3)	O4—C10—C11—C12	91.9 (3)
C4—C5—C6—C7	0.0 (4)	O3—C10—C11—C12	−88.6 (2)
C5—C6—C7—C8	−1.0 (4)	C10—C11—C12—C13	61.4 (2)
C6—C7—C8—C9	0.6 (3)	C10—C11—C12—C15	−60.0 (2)
O2—C4—C9—C8	178.16 (18)	C10—C11—C12—C14	−177.8 (2)

### Hydrogen-bond geometry (Å, º)

**Table:**

*D*—H···*A*	*D*—H	H···*A*	*D*···*A*	*D*—H···*A*
C2—H2···O4	0.93	2.44	2.847 (3)	107
C5—H5···O1^i^	0.93	2.48	3.405 (2)	176

Symmetry code: (i) *x*−1/2, −*y*+1/2, *z*.
